# Diet and Nutraceutical Supplementation in Dyslipidemic Patients: First Results of an Italian Single Center Real-World Retrospective Analysis

**DOI:** 10.3390/nu12072056

**Published:** 2020-07-10

**Authors:** Andrea Pasta, Elena Formisano, Anna Laura Cremonini, Elio Maganza, Erika Parodi, Sabrina Piras, Livia Pisciotta

**Affiliations:** 1 Department of Internal Medicine, University of Genoa, 16132 Genoa, Italy; andreapasta93@gmail.com (A.P.); annalauracremonini@gmail.com (A.L.C.); elio.maganza@gmail.com (E.M.); erika-parodi@alice.it (E.P.); piras.sabri@tiscali.it (S.P.); 2 Nutritional Unit ASL-1 Imperiese, Giovanni Borea Civil Hospital, 18038 Sanremo, Italy; e.formisano@asl1.liguria.it; 3 Dietetics and Clinical Nutrition Unit, IRCCS Policlinic Hospital San Martino, 16132 Genoa, Italy

**Keywords:** lipid lowering diet, nutraceutical supplements, dyslipidemias, hypercholesterolemia, hypertriglyceridemia, EAS/ESC guidelines LDL-C targets

## Abstract

Background: Dyslipidemias are a heterogeneous group of metabolic disorders mainly characterized by an increased risk of atherosclerotic cardiovascular disease (ASCVD) or other conditions, such as acute pancreatitis in hypertriglyceridemia. The aim of this study was to evaluate the effect of diet treatment and nutraceutical (NUTs) supplementation on the plasma lipid profile in outpatient dyslipidemic subjects, considering the influence of several factors (i.e., gender, age, body mass index, alcohol consumption, and smoking habits). Methods: 487 dyslipidemic patients spanning from 2015 to 2019 were treated with a Mediterranean diet or NUTs in a real-word setting and were retrospectively analyzed. General characteristics and lipid profile at baseline and after the follow-up period were evaluated. Results: Diet alone reduced total cholesterol (−19 mg/dL, −7.7%), LDL cholesterol (−18 mg/dL, −10.1%), and triglycerides (−20 mg/dL, −16.7%). Triglycerides (TG) decreased more in men, while women were associated with higher reduction of LDL cholesterol (LDL-C). Different types of NUTs further ameliorate lipid profiles when associated with diet. Nevertheless, most patients at low ASCVD risk (222 out of 262, 81.6%) did not achieve the 2019 ESC/EAS guidelines recommended LDL-C goals (i.e., LDL-C < 116 mg/dL). Conclusion: Lipid-lowering diet improves lipid profile, and NUTs can boost its efficacy, but taken together they are mainly unsatisfactory with respect to the targets imposed by 2019 EAS/ESC guidelines.

## 1. Introduction

Dyslipidemias are a heterogeneous group of metabolic disorders mainly characterized by an increased risk of atherosclerotic cardiovascular disease (ASCVD) [1,2,3]. The most common phenotypes of hyperlipidemias are hypercholesterolemia, characterized by high levels of serum cholesterol due to high low-density lipoprotein cholesterol (LDL-C) levels, hypertriglyceridemia, caused by high levels of triglycerides (TG), and mixed hyperlipidemia, which refers to both elevated LDL-C and plasma TG [4]. Severe hypertriglyceridemia (TG > 1000 mg/dL) is associated with increased risk of acute pancreatitis [5]. Hypoalphalipoproteinemia is directly related to the risk of coronary heart disease [6].

To date the pharmacological treatment of hyperlipidemias involves various types of drugs: statins (inhibitors of cholesterol synthesis) have fully demonstrated efficacy in improving the lipid profile and reducing cardiovascular risk [7], however, LDL-C serum levels achieved with statins do not fully reduce the risk of ASCVD [8]. These drugs can be associated with ezetimibe (inhibitor of cholesterol intestinal absorption) [9,10] or to innovative biological therapies such as monoclonal antibodies [11]. Fibrates are also used in lipid-lowering therapies, especially in patients with high levels of triglycerides [12].

The guidelines for the management of dyslipidemias [13,14] support the role of lifestyle change as a basic treatment. Lifestyle treatment involves a diet based on the Mediterranean model, low in saturated fat and cholesterol and rich in whole grains, vegetables, fruits, and increased physical activity [15,16]. Patients with hypertriglyceridemia should also reduce sugar intake and avoid alcohol consumption [17]. Most evidence has shown that diet treatment has a low and variable effect on serum LDL-C, in a range of 5–15% [18,19,20,21].

Consequently, in clinical practice, the use of diet alone for the treatment of dyslipidemias could be unsatisfactory. In fact, in low- and moderate-risk patients, the 2016 ESC/EAS guidelines [13] recommend LDL-C levels less than 115 mg/dL, but the new 2019 ESC/EAS guidelines [14] recommended LDL-C < 116 mg/dL and < 100 mg/dL in low- and moderate-risk patients, respectively.

As a part of lifestyle modifications, the use of lipid lowering nutraceuticals (NUTs) can be considered alone or in combination with diet and provide a moderate reduction in blood cholesterol [14]. Lipid lowering nutraceuticals are natural dietary supplements which can be used to improve the lipid profile in low ASCVD risk patients and subjects with statin intolerance [22,23]. These compounds act in different ways: monacolin K is an inhibitor of hepatic cholesterol biosynthesis, berberine acts as a promoter of cholesterol uptake in the liver inhibiting the proprotein convertase subtilisin/kexin type 9 (PCSK9) and plant sterols are inhibitors of intestinal cholesterol absorption [24]. Other class of nutraceuticals are represented by Fatty acids-ω3, which are successfully used in the treatment of hypertriglyceridemia [25,26,27].

To date, the factors which could influence the success of a lifestyle intervention with diet alone or supplemented with NUTs are still objects of investigation. Furthermore, an open finding is the contextualization of the improvement of the lipid profile, and in particular of the LDL-C, in the recommendations of the ESC/EAS guidelines for the management of dyslipidemias. Thus, the present study aims to evaluate the effect of diet treatment and NUTs on plasma lipid parameters, and how specific factors (i.e., gender, age, body mass index, alcohol consumption and smoking habits) could influence lipid profile and lifestyle modifications in a real-world setting considering the targets of ESC/EAS guidelines as references.

## 2. Materials and Methods

### 2.1. Subjects

The present study is a retrospective analysis of 2472 patients’ medical records affected by different types of dyslipidemias and referred to the outpatient section of the Lipid Clinic of IRCCS Policlinic San Martino Hospital, University of Genoa, Italy, from 2015 to 2019.

At baseline, all subjects underwent a medical evaluation: family, remote, and near pathological history were considered, and body mass index (BMI) and arterial pressure were assessed. Complete lipid profile, measured in the absence of therapy, was reported (total cholesterol, high-density lipoprotein cholesterol, and TG). A specialized physician with extensive experience in the management of dyslipidemias stratified all patients according to their cardiovascular risk through SCORE algorithm or diagnosing a pathological condition, such as familial hypercholesterolemia, familial combined hyperlipidemia, or other high cardiovascular risk related conditions. The therapeutic strategies were formulated according to the guidelines’ recommendations [13,14] and the rules of the Italian Ministry of Health [28]. Thus, the treatments included lifestyle modifications (i.e., diet and nutraceutical supplements, loss of weight, physical activity, smoking discontinuation) associated or not with lipid-lowering drug therapy. All subjects were expected to return a few months later and underwent a follow-up visit to evaluate the effects of the therapy on biochemical tests. The compliance to the lifestyle intervention was assessed during the follow-up visit by asking the patients.

**Figure 1:**
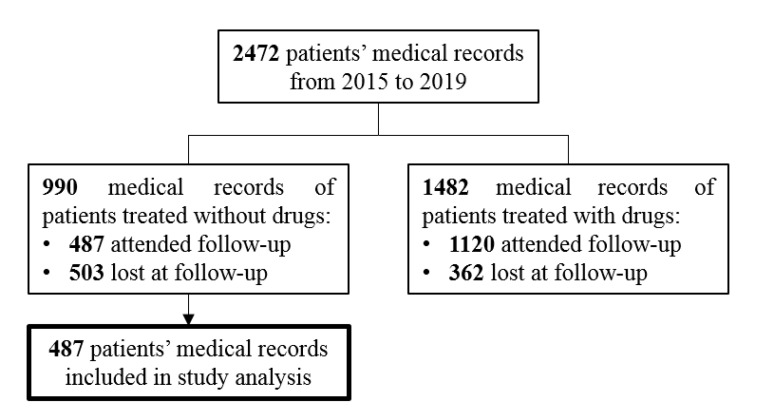
Flow chart of study design and allocation criteria and grouping for patients’ former analysis.

In this retrospective study, we focused our formal analysis on 487 patients treated only with lifestyle modifications coming back at the follow-up visit. The flow chart in Figure 1 reports the allocation criteria and grouping for patients’ analysis as previously described.

Informed written consent for using personal data for the present investigation was obtained from all the subjects. The study was conducted in accordance with the Declaration of Helsinki and was approved by the Ethics Committee of IRCCS Policlinic Hospital San Martino in Genoa (Italy) (Project number 270/2020).

### 2.2. Data Collection and Ranking Methods

Blood tests performed in an authorized laboratory within four weeks since baseline were evaluated and total cholesterol (TC), high-density lipoprotein cholesterol (HDL-C) and TG were retrospectively registered. LDL-C was calculated by the Friedewald formula. The same data were collected at follow-up.

Gender, age, systolic and diastolic blood pression (SBP, DBP), follow-up period, weight, height, and BMI were registered. Patients were classified in no/past smokers and current smokers [29]. Alcohol intake was collected as follow: no/moderate consumption if lower than 10 g/day and excessive consumption if higher than 10 g/day [14]. BMI and age of patients were binary ranked in normal or overweight [30] and in younger or older than 45 years [31], respectively.

### 2.3. Lifestyle Intervention

According to the ESC/EAS guidelines for the management of dyslipidemias [13,14], a lipid lowering diet, based on the Mediterranean model, was administered in all patients and was characterized by a lipid intake between 25 and 35% of the daily kcal, with saturated fats <7% of the total kcal and cholesterol lower than 300 mg/day. The carbohydrate and protein intakes were 45–55% and 15–25% of the total daily kcal, respectively. Patients were also advised to do regular moderate-intensity exercise (≥30 min a day) for both normal-weight and overweight.

After the evaluation of anthropometric and hematochemical parameters, four different types of diets were administered in an outpatient setting:

- General advice to reduce the excess of saturated fats and cholesterol intake based on weekly food frequency in normal weight patients with primary hypercholesterolemia
- General advice to reduce the excess of carbohydrates and alcohol based on weekly food frequency in normal weight patients with hypertriglyceridemia or mixed hyperlipemia
- Diet with weekly food frequency and total energy intake of 1700 kcal/day in overweight women
- Diet with weekly food frequency and total energy intake of 2100 kcal/day in overweight men.

NUTs were administered in some subjects according to guidelines: Monacolin K (MonK), Phytosterols (PS), Berberine (BBR), Omega-3 Polyunsaturated Fatty Acids (PUFA-W3) and associations of them (i.e., Monacolin K, 3 mg or 10 mg + Berberine 500 mg, Monacolin K + Fatty acids - ω3).

Detailed diet information is reported in supplementary material, while the comprehensive diet composition is shown in Supplementary Table S1.

### 2.4. Statistical Analysis

Statistical analyses were performed using SPSS Statistics 25, Release Version 25.0; SPSS, Inc., 2017, Chicago, IL, USA (www.spss.com). Detailed statistical analysis is reported in the supplementary material.

## 3. Results

### 3.1. Baseline Population Characteristics

The flow chart in Figure 1 showed that patients on statin therapies returned to the follow-up visit more frequently than patients who received the diet alone (1120, 75.6% vs. 487, 49.2%, respectively) at Person’ chi square test (*p* < 0.0001).

The clinical characteristics of all 487 subjects on the diet treatment with or without NUT supplementation, 201 men and 286 women, included in the main analysis were reported in Table 1. Most of the patients were natives of the North-West region of Italy, while only three patients were born in the Middle East (0.6%) and four subjects in South America (0.8%). All the subjects included lived in the Liguria region. The median follow-up period was 4 months (IQR 3–10 months) in patients treated with diet alone and five months (IQR 3–11 months) in subjects supplemented with NUT. No statistically significant differences have been observed among patients divided in the two different lifestyle modification approaches (i.e., Diet alone and diet plus NUT).

The differences between male and female gender in age, anthropometric measures, and smoking and alcohol consumption have been analyzed: men were younger than females, and males’ weight and BMI were higher and tobacco and alcohol consumption (>10 g/day) was more frequent than in women (comprehensive characteristics have been reported in supplementary Table S2).

The baseline lipid profile of 487 subjects and the differences analyzed within patients divided according to gender, age, BMI, smoking habit, alcohol consumption, and treatment with or without NUT were reported in Supplementary Table S3. Females had a higher level of TC and HDL-C and a lower level of TG than males. HDL-C was significantly lower in younger subjects (age <45 years) than older ones. In overweight patients HDL-C and TG were respectively lower and higher than normal weight patients. No significant differences were observed in subgroups for LDL-C. Cross-sectional analyses were also performed of the relationships between the lipid profile and other variables included in the baseline study (Supplementary Table S4). The presence of lower TC and HDL-C and higher TG were independently correlated with male gender. Furthermore, higher HDL-C was independently correlated with normal weight patients while TG was independently correlated with overweight patients (Supplementary Table S4).

### 3.2. Effect of Diet Alone Treatment

Between the baseline and follow-up, the median values of TC, LDL-C, and TG decreased by −7.7%, −10.1%, and −16.7%, respectively, in the 207 patients treated with diet alone. No significant variation was observed in the HDL-C value. A similar improvement in the lipid profile was observed in all analyzed subgroups of patients, however females were characterized by a larger reduction of LDL-C than males. Conversely, triglycerides decreased more in men than in women and in overweight than in normal weight patients (Table 2). A multivariate analysis (see statistical analysis for detailed formal description) showed a positive linear correlation between higher reduction in LDL-C and female gender and, on the other hand, a higher reduction in TG and male gender (Supplementary Table S5).

### 3.3. Effect of Lipid Lowering Nutraceuticals

**Figure 2:**
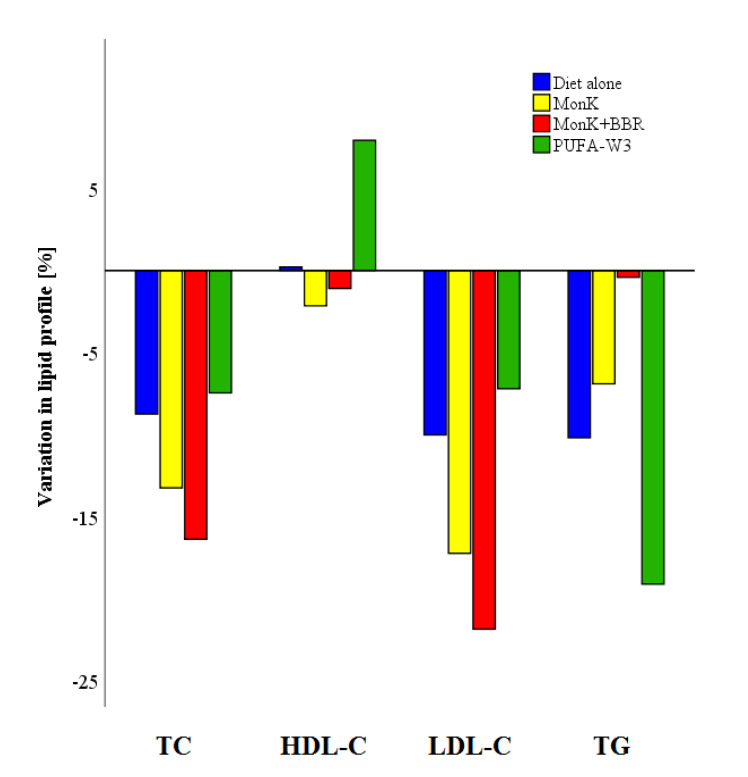
Percentage variation of lipid parameters in different NUTs groups.

Overall, 280 subjects underwent lipid lowering treatment with diet plus NUT. General characteristics and lipid parameters have been reported in supplementary Table S6. However, formal analysis considered only patients in three therapy groups: (1) 29 subjects with MonK (3 mg/day or 10 mg/day), (2) 167 subjects with Monk (3 mg/day or 10 mg/day) plus BBR (500 mg/day), and (3) 36 patients with PUFA-W3 (3 g/day). Supplements used in less than 11 subjects have been excluded as well as products with uncertain component titration (i.e., pharmacy galenic formulas). Variation in lipid parameters between baseline and follow-up within the three groups have been reported in Table 3, while medians of percentage variations have been shown in Figure 2. Moreover, the differences in median values have been calculated within NUT groups and diet alone (Table 3). The most effective NUTs group in reducing LDL-C was MonK plus BBR, which was also more effective than PUFA Omega 3 (*p* < 0.0001) and MonK alone (*p* = 0.294, NS). TG reduction was greater in patients treated with PUFA Omega 3 compared to diet alone (*p* < 0.049), MonK (*p* = 0.058, NS) and MonK plus BBR (*p* < 0.0001).

### 3.4. Lipid Lowering Intervention and Therapeutic Targets

Patients at baseline visit were stratified according to their ASCVD risk through SCORE algorithm or diagnosing a pathological condition, such as familial hypercholesterolemia, familial combined hyperlipidemia or other ASCVD risk related conditions [13,14]. Table 4 reports the different success of our non-statin treatment (i.e., diet and diet plus NUT) in reaching 2016 and 2019 ESC/EAS LDL-C targets. At follow-up, the goals of the 2016 ESC/EAS guidelines were achieved in 40/262 (15.3%) and 23/170 (13.5%) respectively in low and moderate ASCVD risk (recommended LDL-C <115 mg/dL). However, 40/262 (15.3%) and 11/170 (6.5%) patients reached the 2019 ESC/EAS recommended LDL-C values in low (LDL-C≤115 mg/dL) and moderate (LDL-C<100 mg/dL) ASCVD risks. Only 3 out of 45 high-risk patients reached the 2016 ESC/EAS recommended LDL-C values, and none of the very-high risk subjects (n. 10) reached the values. Conversely, no patients in high or very-high ASCVD risk classes reached the 2019 ESC/EAS recommended LDL-C goals.

On the other hand, lifestyle modification successfully increased the percentage of patients reaching LDL-C goals after the follow-up period (Table 4).

## 4. Discussion

The main purpose of this study was to evaluate the effect of diet treatment and NUTs on plasma lipid parameters in a real-world setting, taking into account possible influencing factors (i.e., gender, age, BMI, alcohol consumption, and smoking habits). The recommended lipid goals of the guidelines for the management of dyslipidemias have been used as a reference to estimate the clinical efficacy of administered interventions.

A preliminary result is the higher percentage of pharmacologically-treated patients (i.e., with statins, ezetimibe, etc.) who returned to the follow-up visit compared to patients who received only the lifestyle intervention. Some studies reported that the adherence to lifestyle intervention was unsatisfactory and significantly lower than in patients treated with drugs [32,33,34]. Thus, our results may be related to patients’ altered behavior between diet and drugs and our study cases probably considered drug therapy more effective and important than lifestyle intervention. Thus, patients treated only with lifestyle intervention returned less frequently to the follow-up visit and may have considered lifestyle intervention as a second-rate therapy.

An important result of the study was the significant reduction of TC (−19 mg/dL, −7.7%), LDL-C (−18 mg/dL, −10.1%) and TG (−20 mg/dL, −16.7%) with the administration of a diet alone treatment. We observed an improvement of the lipid profile, which was consistent with the literature despite the lifestyle intervention having been conducted in an outpatient setting and the dietitian counseling lasting only 10 min on average. An important position paper of an International Lipid Expert Panel reported an LDL-C reduction between 5% and 15% with lifestyles interventions based on the Mediterranean diet [24]. Likewise, there are no unequivocal data on the reduction of TG that is reported between 5% and 20% [35], but with important differences among subjects.

Actually, some cross-sectional studies reported that the female gender is associated with higher HDL-C and lower TG levels [36,37] but female adherence to lifestyle modifications is higher than men [38]. The present data showed that the characteristics of the baseline lipid profile and the reduction of LDL-C in males and females agreed with the literature, except for TG levels, which decreased more in men. The differences in the basal lipid profile and in the dietary effects could be related to specific and not quantifiable traits of male gender (i.e., psychological, social, and cultural patterns) but also to a worse anthropometric and lifestyle features observed in our study cases. Men were found to have higher BMI and alcohol consumption and worse smoking habits than women. This phenotypic characteristic is consistent with literature: hypertriglyceridemia was related to high alcohol intake, obesity [39], and male gender [40], while low HDL-C levels were also associated with smoking habits [41], obesity, and male gender. On the other hand, the larger decrease in TG observed in men was probably due to the higher basal levels in comparison to women in which levels were substantially normal.

Subjects who received NUTs have been characterized by a greater reduction of lipid parameters than from diet alone. In particular, the LDL-C and TC reduction with MonK was −14.7% and −11.3%, respectively, while other studies reported a percentage variation ranging from −27.3% to −12.5% and from −15.5% to −6.6% in LDL-C and TC, respectively [42,43,44,45,46,47]. Noteworthy, the combination of MonK and BBR was the most effective NUT in reducing LDL-C and TC (−23.4% and −17.4%, respectively). This finding is consistent with literature [24,48] and could be useful in patients with low ASCVD risk, even if the guidelines for the management of dyslipidemias highlighted the lack of high-quality randomized clinical trials. Furthermore, this type of NUTs as an addition to the diet could have various beneficial effects on the risk of atherosclerosis not only related to LDL-C reduction, as previously documented in some publications by other groups and ours [49,50]. On the other hand, the diet supplemented with PUFA-W3 was the most effective TG lowering treatment with a median percentage reduction of −22.6% from baseline to follow-up. This outcome agreed with a recent metanalysis which showed the efficacy of 3 g/day of PUFA-W3 in reducing TG of about 14% [51].

Another relevant finding emerging from our study was the poor success in our patients with both lifestyle interventional strategies (i.e., diet alone and diet plus NUT) to reach the new LDL-C goals recommended by ESC/EAS guidelines. In fact, after the follow-up period, only 40 (15. 3%) and 11 (6.5%) subjects out of 262 and 170 in low and moderate ASCVD risk, respectively, were at ESC/EAS 2019 [14] targets and no patients in high and very-high risk classes reached the targets. The results obtained considering the 2016 targets were comparable with the reported analysis of the 2019 targets. Only a few more patients at moderate and high ACSVD risk achieved the less ambitious LDL-C targets of 2016 ESC/EAS guidelines (n. 23 with LDL-C <115 mg/dL vs. n. 11 with LDL-C<100 mg/dL in moderate risk class and n. 3 with LDL-C <100 mg/dL vs. n. 0 with LDL-C <70 mg/dL in high risk class, respectively).

All patients not reaching LDL-C goals should undergo administration of a pharmacological lipid-lowering drug such as statins. The latter is undoubtedly the best clinical strategy in patients at moderate to very-high ASCVD risk, except for contraindications. However, in our analysis, the patients at high and very-high ASCVD risk were not treated with drugs at baseline due to clinical history of statin intolerance or patients’ decision, despite the guideline’s recommendation. In low ASCVD risk subjects, different from the 2016 ESC/EAS guidelines which did not recommended pharmacological therapy if LDL-C<190 mg/dL, the 2019 ESC/EAS guidelines recommend also the use of drug therapy when lifestyle intervention was unsatisfactory in reducing LDL-C levels of at least 50% and less than 116 mg/dL (Class IIb and level of evidence A). However, the scientific literature is not conclusive on the need for statins in persons at low risk of ASCVD [52]. The reasons may be the high number of patients needed to treat to avoid nonfatal ASCVD (1:217 for myocardial infarction and 1:313 for stroke) [53], the risk of new onset type 2 diabetes mellitus [54] and the risk of statin-related side effects, such as musculoskeletal pain, elevation in creatine phosphokinase and hepatic dysfunctions [55]. Moreover, the Italian Ministry of Health authorized the refund of statins and other lipid-lowering drugs only for patients with ASCVD risk higher than 2% [28]. Finally, the patients belonging to the low-risk class who did not reach LDL-C targets were mainly woman (n. 130/222, 58.6%), non-smokers (n. 190/222, 85.6%), normotensives (median SBP 126, IQR 124–127 and median DBP 84, IQR 81–86) and relatively younger patients (median 43, IQR 36–50 years). In our clinical experience, the low-risk patients with such described clinical characteristics are often resistant to accept pharmacological treatments. This observation is supported by literature which reported that the young age, the female gender and the absence of cardiovascular risk factors were associated with less adherence and prescription of statin therapy [56].

An important limitation of this study is represented by the relatively small sample size analyzed. This limit may have interfered with the possibility of carrying out more subgroup analyzes with greater statistical power. In light of this issue, we believe that new studies are necessary to confirm our results and we will consider the increasing of the patients’ size as a priority in the near future. Another limitation of our study is the lack of the use of objective methods (i.e., food frequency questionnaire, food diary) to assess the compliance to the lifestyle intervention.

## 5. Conclusions

A lipid-lowering diet based on Mediterranean models improves lipid profiles in an outpatient real-world setting. The addition of NUTs can boost dietary efficacy, in particular MonK and its association with Berberine reduce LDL-C more than diet, while the larger improvement of triglyceridemia has been recorded with PUFA-W3 supplementation. However, all lifestyle modifications were mainly unsatisfactory with respect to the targets imposed by EAS/ESC guidelines, but the pharmacological treatment with statins in patients with low risk of ASCVD is still under discussion. Further studies will be needed to confirm our results and optimize the treatment of lipid risk factors, particularly in patients with low ASCVD risk.

## Figures and Tables

**Table 1:** Baseline characteristics of 487 dyslipidemic non-statin treated patients divided in diet alone and diet plus NUT groups.

Variable	Diet Alone	Diet + Nut
Sex [F/M: n; %]	117 (56.5%)/90 (43.5%)	169 (60.4%)/111 (39.6%)
Age		
[years: mean±SD; median; IQ range]	50 ± 15; 51 (40, 60)	52 ± 13; 52 (43, 61)
[<45 years/≥45 years: n, %]	78 (37.7%)/129 (62.3%)	88 (31.4%)/192 (68.6%)
Weight		
[kg: mean±SD; median; IQ range]	71.0 ± 15.5; 69.0 (60.0, 81.0)	69.2 ± 14.3; 69.7 (60.0, 77.0)
BMI		
[kg/m^2^: mean±SD; median; IQ range]	25.2 ± 4.3; 24.7 (22.1, 28.0)	24.8 ± 3.8; 24.3 (22.5, 26.8)
[<25 kg/m^2^/≥25 kg/m^2^]	110 (53.1%)/97 (46.9%)	166 (59.3%)/114 (40.7%)
SBP		
[mm/Hg: mean ± SD; median; IQ range]	126 ± 2; 126 (125, 128)	127 ± 2; 127 (125, 128)
DBP		
[mm/Hg: mean ± SD; median; IQ range]	85 ± 3; 85 (83, 87)	85 ± 3; 85 (83, 87)
Risk SCORE		
[%: mean ± SD; median; IQ range]	2.1 ± 3.0; 0.9 (0.3, 2.9)	2.0 ± 2.9; 0.9 (0.3, 2.5)
Low-Risk: <1% [n; %]	117 (56.5%)	145 (51.8%)
Moderate-Risk: ≥1% and <5% [n; %]	64 (30.9%)	106 (37.9%)
High-Risk: ≥5% and <10% [n; %]	22 (10.6%)	23 (8.2%)
Very-High-Risk: ≥10% [n; %]	4 (1.9%)	6 (2.1%)
Smoking habits		
[Never + Past/Current: n; %]	155 (74.9%)/52 (25.1%)	218 (77.9%)/62 (22.1%)
Alcohol intake		
[No + Moderate/Excessive: n; %]	134 (65.0%)/72 (35.0%)	208 (74.8%)/70 (25.2%)

Abbreviations: M = male, F = female, y = years, BMI = body mass index, SBP = systolic blood pressure, DBP = diastolic blood pressure, NUT = lipid lowering nutraceutical.

**Table 2:** Effect of diet alone on lipid profile overall and in specific groups of patients.

	TC			HDL-C			LDL-C			TG		
	Baseline (Median, IQR)	Follow-Up (Median, IQR)	Variation † (Median, IQR, %, *p*-Value)	Baseline (Median, IQR)	Follow-Up (Median, IQR)	Variation † (Median, IQR, %, *p*-Value)	Baseline (Median, IQR)	Follow-Up (Median, IQR)	Variation † (Median, IQR, %, *p*-Value)	Baseline (Median, IQR)	Follow-Up (Median, IQR)	Variation † (Median, IQR, %, *p*-Value)
**All patients** (n:207)	259 (241, 285)	237 (205, 260)	−19 (−46, −6) −7,7%, *p* < 0.001	54 (42, 66)	53 (42, 66)	0 (−6, 5) 0.0%, NS	176 (154, 194)	155 (125, 177)	−18 (−40, −1) −10.1%, *p* < 0.001	142 (96, 232)	120 (82, 173)	−20 (−68, 7) −16,7%, *p* < 0.001
Sex *												
F (n: 117, 56.5%)	264 (250, 287)	241 (216, 261)	−22 (−54, −10) −8.4%, *p* < 0.001	61 (50, 73)	62 (48, 73)	1 (−6, 5) 0.7%, NS	178 (159, 192)	157 (128, 176)	−20 (−45, −7) −11.4%, *p* < 0.001	110 (87, 168)	100 (76, 163)	−13 (−44, 9) −12.2%, *p* < 0.001
M (n: 90, 43.5%)	250 (222, 275)	224 (203, 256)	−15 (−39, 2) −6.6%, *p* < 0.001	47 (37, 56)	45 (39, 54)	0 (−6, 5) 0.0%, NS	169 (137, 196)	153 (122, 178)	−8 (−30, 8) −5.6%, *p* = 0.001	197 (130, 315)	136 (105, 189)	−45 (−150, −2) −27.2%, *p* < 0.001
Age *												
<45 years (n: 78, 129%)	256 (222, 284)	228 (200, 256)	−20 (−45, −4) −7.7%, *p* < 0.001	53 (41, 63)	52 (41, 63)	0 (−7, 5) 0.0%, NS	170 (152, 192)	151 (117, 174)	−17 (−41, 3) −9.9%, *p* < 0.001	154 (101, 229)	124 (80, 172)	−20 (−73, 8) −14.9%, *p* < 0.001
≥45 years (n: 129, 62.3%)	261 (246, 287)	240 (212, 261)	−19 (−49, −8) −7.7%, *p* < 0.001	54 (43, 67)	55 (45, 70)	1 (−6, 5) 1.6%, NS	177 (158, 196)	158 (130, 178)	−18 (−40, −4) −10.1%, *p* < 0.001	136 (96, 235)	120 (82, 174)	−21 (−67, 2) −18.2%, *p* < 0.001
**BMI**												
<25 kg/m^2^ (n: 110, 53.1%)	257 (242, 281)	236 (205, 256)	−20 (−49, −6) −7.5%, *p* < 0.001	59 (49, 70)	59 (47, 68)	0 (−7, 5) 0.0%, NS	174 (155, 190)	152 (122, 175)	−19 (−40, −3) −11.1%, *p* < 0.001	117 (82, 180)	101 (77, 154)	−11 (−47, 11) −11.1%, *p* < 0.001
≥25 kg/m^2^ (n: 97, 46.9%)	261 (238, 288)	239 (206, 263)	−17 (−45, −6) −8.1%, *p* < 0.001	48 (40, 60)	47 (40, 63)	1 (−5, 6) 2.2%, NS	179 (154, 198)	158 (130, 179)	−16 (−40, 1) −9.1%, *p* < 0.001	196 (120, 315)	137 (99, 209)	−40 (−121, −7) −23.5%, *p* < 0.001
Smoking habits *												
Never/Past (n: 155, 74.9%)	262 (241, 287)	240 (205, 261)	−19 (−44, −6) −7.5%, *p* < 0.001	54 (44, 66)	54 (42, 67)	0 (−6, 5) 0.0%, NS	177 (155, 197)	156 (127, 179)	−17 (−38, −1) −9.9%, *p* < 0.001	138 (94, 228)	119 (81, 168)	−18 (−63, 8) −14.5%, *p* < 0.001
Current (n: 52, 25.1%)	257 (237, 277)	227 (206, 251)	−19 (−52, −7) −8.2%, *p* < 0.001	53 (40, 65)	52 (44, 64)	2 (−6, 10) 4.7%, NS	171 (152, 187)	153 (121, 172)	−18 (−45, 1) −11.0%, *p* < 0.001	161 (102, 320)	123 (88, 185)	−37 (−147, −2) −26.3%, *p* < 0.001
Alcohol consumption *												
Absent/moderate (135, 64.3%)	262 (237, 288)	234 (205, 260)	−22 (−53, −6) −8.4%, *p* < 0.001	53 (41, 66)	54 (42, 67)	1 (−4, 6) 2.6%, NS	179 (155, 194)	154 (121, 176)	−20 (−45, −2) −11.4%, *p* < 0.001	143 (96, 235)	115 (81, 168)	−20 (−68, 8) −16.2%, *p* < 0.001
Elevate (72, 35.7%)	257 (242, 278)	237 (208, 262)	−16 (−40, −4) −6.0%, *p* < 0.001	54 (46, 65)	52 (43, 64)	−1 (−7, 4) −2.1%, NS	170 (152, 194)	157 (133, 178)	−16 (−29, 8) −8.4%, *p* = 0.01	134 (96, 229)	132 (82, 177)	−19 (−67, 6) −18.2%, *p* < 0.001

Plasma lipid concentrations are reported in mg/dL or percentage of variation. Abbreviations: M = male, F = female, BMI = body mass index, IQR, interquartile range, * Independent pairwise comparisons among subgroup terms with Mann-Whitney U test adjusted for multiple comparisons (Bonferroni). † *p*-values for dependent samples nonparametric Wilcoxon Signed Ranks Test between baseline and follow-up values. The difference is significant at the 0.001 level between subgroup terms (i.e., male vs female and normal weight vs overweight).

**Table 3:** Effect of different groups of NUTs on lipid profile and comparison with diet alone by the difference in the median.

	MonK (n. 29)	MonK + BBR (n. 167)	PUFA-W3 (n. 36)	Bonferoni adj. *p*-Value † (Unadjusted)
TC (mg/dL)	−30 (−48; −20),	−46 (−66; −23),	−15 (−37; 5),	MonK vs. PUFA-W3: 0.086 (0.014) MonK + BBR vs. Diet: <0.0001 (<0.001) MonK + BBR vs. PUFA-W3: <0.0001 (<0.0001)
(median, IQR, %)	−11,3%	−17,4%	−6%	
**Dif. from diet alone**				
(median, %)	−11, −3,6%	−27, −9,7%	4, 1,7%	
HDL-C (mg/dL)	−1 (−6; 1),	0 (−7; 5),	3 (−1; 7),	NS
(median, IQR, %)	−2,2%	0%	5,3%	
**Dif. from diet alone**				
(median, %)	−1, −2,2%	0, 0%	3, 5,3%	
LDL-C (mg/dL)	−23 (−41; −14),	−42 (−62; −20),	−11 (−40; 9),	MonK vs. Diet: 0.294 (0.049) MonK + BBR vs. Diet: <0.0001 (<0.001) MonK + BBR vs. PUFA-W3: <0.0001 (<0.0001) MonK vs. PUFA-W3: 0.253 (0.042)
(median, IQR, %)	−14,7%	−23,4%	−7,2%	
**Dif. from diet alone**				
(median, %)	−5, −4,6%	−24, −13,3%	7, 2,9%	
TG (mg/dL)	−12 (−39; 1),	−11 (−43; 16),	−66 (−148; −18),	PUFA-W3 vs. Diet: 0.049 (0.008) MonK vs. PUFA-W3: 0.058 (0.010) MonK + BBR vs. PUFA-W3: <0.0001 (<0.0001) MonK + BBR vs. Diet: 0.027 (0.004)
(median, IQR, %)	−14,1%	−9,4%	−22,6%	
**Dif. from diet alone**				
(median, %)	8, 2,6%	9, 7,3%	−46, −5,9%	

Data are variation of lipid profile variable and are presented as median, IQR and percentage of variation. Difference in median from diet alone are also shown. Abbreviations: MonK = monacolin (10 mg/day), MonK + BBR = monacolin (3–10 mg/day) plus berberine (500 mg/day), PUFA-W3= Ω3 − polyunsaturated fatty acid. † Indipendent samples Kruskal–Wallis tests. Significance values have been adjusted by the Bonferroni correction for multiple tests. Unadjusted *p*-values have been also reported.

**Table 4:** Patients reaching and not reaching 2016 and 2019 ESC/EAS LDL-C recommended target in the different risk classes.

ASCVD Risk		Low (<1%)	Moderate (≥1%; <5%)	High (≥5%; <10%)	Very High (≥10%)
2016 ESC/EAS LDL-C targets		<115 mg/dL	<115 mg/dL	<100 mg/dL	<70 mg/dL
	Baseline				
Reaching 2016 ESC/EAS Guidelines	n (%)	8 (3.1%)	7 (4.1%)	0 (0%)	0 (0%)
	F/M	3/5	3/4	NA	NA
	Age (years)	40 (33–45)	64 (57–68)	NA	NA
Not reaching 2016 ESC/EAS Guidelines	n (%)	254 (96.9%)	163 (95.9%)	45 (100%)	10 (100%)
	F/M	150/104	104/59	22/23	4/6
	Age (years)	43 (36–50)	59 (55–65)	68 (64–74)	74 (73–77)
	Follow-up				
Reaching 2016 ESC/EAS Guidelines	n (%)	40 (15.3%)	23 (13.5%)	3 (6.7%)	0 (0%)
	F/M	23/17	16/7	2/1	NA
	Age (years)	41 (33–49)	61 (58–65)	74 (69–77)	NA
Not reaching 2016 ESC/EAS Guidelines	n (%)	222 (84.7%)	147 (86.5%)	42 (93.3%)	10 (100%)
	F/M	130 (58.6%)	91 (61.9%)	20 (46.5%)	4 (40.0%)
	Age (years)	43 (36–50)	59 (55–65)	68 (64–73)	74 (73–77)
2019 ESC/EAS LDL-C targets		<116 mg/dL	<100 mg/dL	<70 mg/dL	<55 mg/dL
	Baseline †				
Reaching 2019 ESC/EAS Guidelines	n (%)	8 (3.1%)	1 (0.6%)	0 (0%)	0 (0%)
	F/M	3/5	1/0	NA	NA
	Age (years)	40 (33–45)	70	NA	NA
Not reaching 2019 ESC/EAS Guidelines	n (%)	254 (96.9%)	169 (99.4%)	45 (100%)	10 (100%)
	F/M	150/104	106/63	22/23	4/6
	Age (years)	43 (36–50)	59 (55–65)	68 (64–74)	74 (73–77)
	Follow-up				
Reaching 2019 ESC/EAS Guidelines	n (%)	40 (15.3%)	11 (6.5%)	0 (0%)	0 (0%)
	F/M	23/17	9/2	NA	NA
	Age (years)	41 (33–49)	62 (58–65)	NA	NA
Not reaching 2019 ESC/EAS Guidelines	n (%)	222 (84.7%)	159 (93.5%)	45 (100%)	10 (100%)
	F/M	130/92	98/61	22/23	4/6
	Age (years)	43 (36–50)	59 (55–65)	68 (64–74)	74 (73–77)

Significant difference (*p* < 0.0001) at Pearson’s chi-squared test vs. follow-up within patient’s ASCVD risk distribution., † Significant difference (*p* < 0.0001) at Pearson’s chi-squared test vs. follow-up within patient’s ASCVD risk distribution.

## Supplementary Materials

The following are available online at https://www.mdpi.com/2072-6643/12/7/2056/s1, Table S1: Composition of diets for overweight patients, Supplementary material 1.1: General advices on weekly food frequency in normal weight patients with primary hypercholesterolemia, Supplementary material 1.2: General advices on weekly food frequency with low carbohydrate intake in normal weight patients with hypertriglyceridemia or mixed hyperlipemia, Supplementary material 1.3: Diet with weekly food frequency and total energy intake of 1700 kcal/day in overweight women, Supplementary material 1.4: Diet with weekly food frequency and total energy intake of 2100 kcal/day in overweight men, Statistical analysis, Supplementary table S2.: General characteristics of patients according to intervention types and gender, Supplementary table S3: Baseline lipid profile in all patients and divided in subgroups according to sex, age, BMI, smoking habits, alcohol consumption and types of lifestyle intervention, Supplementary table S4: Multivariate analysis on baseline lipid profile in all 487 patients, Supplementary table S5: Multivariate analysis between baseline and follow-up of lipid profile in 207 patients on diet alone treatment, Supplementary table S6: General characteristics and lipid parameters of 280 patients on diet plus NUT treatment.

## Abbreviations

ASCVD	atherosclerotic cardiovascular disease
LDL-C	low-density lipoprotein cholesterol
TC	total cholesterol
HDL-C	high-density lipoprotein cholesterol
TG	triglycerides
PCSK9	proprotein convertase subtilisin/kexin type 9
BMI	body mass index
NUT	lipid lowering nutraceutical
MonK	Monacolin K
PS	Phytosterols
BBR	Berberine
PUFA-W3	Fatty acids - ω3
n	number
