# Supplementary material for: Diet and Nutraceutical Supplementation in Dyslipidemic Patients: First Results of an Italian Single Center Real-World Retrospective Analysis
Source: Nutrients. 2020 Jul 10;12(7):2056. doi: 10.3390/nu12072056 (PMC7400882; doi:10.3390/nu12072056)
Supplement: Supplementary file 1 [file nutrients-12-02056-s001.pdf]

SUMMARY

Supplementary Table S1. Composition of diets for overweight patients. 2

1.1 General advices on weekly food frequency in normal weight patients with primary hypercholesterolemia 3

1.2 General advices on weekly food frequency with low carbohydrate intake in normal weight patients with hypertriglyceridemia or mixed hyperlipemia 4

1.3 Diet with weekly food frequency and total energy intake of 1700 kcal/day in overweight women 5

1.4 Diet with weekly food frequency and total energy intake of 2100 kcal/day in overweight men 6

2.4 Statistical analysis 8

Supplementary table S2. General characteristics of patients according to intervention types and gender. 9

Supplementary table S3. Baseline lipid profile in all patients and divided in subgroups according to sex, age, BMI, smoking habits, alcohol consumption and types of lifestyle intervention. 10

Supplementary table S4. Multivariate analysis on baseline lipid profile in all 487 patients 11

Supplementary table S5. Multivariate analysis between baseline and follow-up of lipid profile in 207 patients on diet alone treatment. 12

Supplementary table S6. General characteristics and lipid parameters of 280 patients on diet plus NUT treatment. 13

Supplementary Table S1. Composition of diets for overweight patients.

**NUTRIENTS**	1700 Kcal diet for females	2100 Kcal diet for males
**Proteins** [g, % of total daily energy intake]	72.7 g (17.4%)	92.3 g (17.8%)
**Fats** [g, % of total daily energy intake]	58.6 g (31.5%)	72.9 g (31.5 %)
**Carbohydrates** [g, % of total daily energy intake]	213,5 g (51.1%)	263.5 g (50.7%)
**Sugars** [g/day]	61,4 g	65.7 g
**Fiber** [g/day]	24.0 g	26.2
**Cholesterol** [mg/day]	136,9 mg	159.5 mg
**Saturated Fats** [g, % of total daily energy intake]	10,2 g (5.5%)	12.4 g (5.4%)

1.1 General advices on weekly food frequency in normal weight patients with primary hypercholesterolemia

1. Limit meat consumption twice a week and prefer white meat (i.e. poultry or lean pork), deprived of skin and visible fat. Limit red meat consumption once a week. Avoid offal consumption (i.e. brain and kidney).
2. Increase fish products consumption at least 2-4 times a week; prefer fish rich in omega-3 fatty acids (sardines, herring, anchovies, salmon). Crustaceans and mollusks are not contraindicated because they contain moderate amounts of cholesterol which are lower than meat and cheese.
3. Limit processed meat consumption once a week and prefer hams deprived of visible fat and turkey. Avoid sausages, bacon, salami and corned beef consumption.
4. Limit low-fat cheeses and dairy products consumption twice a week. Grated Parmesan should be limited to 1 tablespoon per day.
5. We recommend daily use of partially skimmed or skimmed milk and yogurt.
6. Limit eggs consumption once a week.
7. Avoid butter, margarine, lard, palm oil and foods that contain them. Use daily extra virgin olive oil daily. Avoid also fried foods, mayonnaise and pre-cooked frozen food.
8. Eat daily bread, pasta, rice, or other cereals, preferably whole meal. Avoid consumption of fresh egg pasta, oil or milk bread and focaccia.
9. Consume legumes at least 2-3 times a week (beans, chickpeas, lentils, peas, broad beans).
10. Consume vegetables and vegetables of any kind daily. Potatoes are not considered vegetables; they are substitutes of bread and pasta and must be consumed no more than once a week due to the high glycemic index.
11. Consume fruit daily. Limit frequency of consumption of very ripe and sugary fruits (i.e. bananas, persimmons, figs, prickly pears, grapes). Avoid coconut for saturated fat content.
12. Consume a small portion of dried nuts daily (walnuts, almonds, hazelnuts, cashews, pistachios).
13. Limit simple sugars consumption at breakfast (i.e. sugar, honey, jam). Limit sugar to 2 teaspoons per day. Avoid biscuits, ice cream, pastry desserts and milk or white chocolate. Without a calorie limit, it is possible to consume homemade desserts occasionally, prepared with extra virgin olive oil, yogurt or partially skimmed milk. Extra dark chocolate is allowed in limited quantities.
14. Alcohol consumption is not recommended. Avoid sugar drinks, industrial teas and fruit juices with or without added sugar.
15. 2-3 cups of coffee per day are allowed.
16. Recommended cooking methods: grilled, grilled, steamed, boiled, roasted, in foil, in the oven. To season the dishes, we recommend to use vegetable or meat broth, tomato sauce, vegetables, spices such as basil, oregano, saffron, marjoram, pepper, garlic.
17. Limit use of salt and avoid all foods rich in salt (pickled or canned foods).
18. It is recommended to do regular moderate-intensity exercise ($\geq$ 30 minutes a day)

1.2 General advices on weekly food frequency with low carbohydrate intake in normal weight patients with hypertriglyceridemia or mixed hyperlipemia

1. Limit meat consumption twice a week and prefer white meat (i.e. poultry or lean pork), deprived of skin and visible fat. Limit red meat consumption once a week. Avoid offal consumption (i.e. brain and kidney).
2. Increase fish products consumption at least 2-4 times a week; prefer fish rich in omega-3 fatty acids (sardines, herring, anchovies, salmon). Crustaceans and mollusks are not contraindicated because they contain moderate amounts of cholesterol which are lower than meat and cheese.
3. Limit processed meat consumption once a week and prefer hams deprived of visible fat and turkey. Avoid sausages, bacon, salami and corned beef consumption.
4. Limit low-fat cheeses and dairy products consumption twice a week. Grated Parmesan should be limited to 1 tablespoon per day.
5. We recommend daily use of partially skimmed or skimmed milk and yogurt.
6. Limit eggs consumption once a week.
7. Avoid butter, margarine, lard, palm oil and foods that contain them. Use daily extra virgin olive oil daily. Avoid also fried foods, mayonnaise and pre-cooked frozen food.
8. Eat daily bread, pasta, rice, or other cereals, preferably whole meal. Avoid consumption of fresh egg pasta, oil or milk bread and focaccia.
   Bread portion: 100 g per day (or whole meal bread: 130 g per day) pasta portion: 80 g per day.
9. Consume legumes at least 2-3 times a week (beans, chickpeas, lentils, peas, broad beans).
10. Consume vegetables and vegetables of any kind daily. Potatoes are not considered vegetables; they are substitutes of bread and pasta and must be consumed no more than once a week due to the high glycemic index. Vegetable portion: free. Potato portion: 350 g instead of 80 g of pasta or 100 g of bread.
11. Consume fruit daily. Limit frequency of consumption of very ripe and sugary fruits (i.e. bananas, persimmons, figs, prickly pears, grapes). Avoid coconut for saturated fat content.
    Fruit portion: two per day.
    Portion of pineapple oranges, cherries, kiwi, mapo, pears, apples, plums: 150 g
    Portion of apricots, watermelon, strawberries, raspberries, quinces, melon, blueberries, blackberries, medlars, peaches, grapefruit: 210 g
    Fruits to be limited: bananas, persimmons, figs, prickly pears, mandarins, mangoes, pomegranates, grapes: 100 g
12. Consume a small portion of dried nuts daily (walnuts, almonds, hazelnuts, cashews, pistachios). Portion: 20 g
13. Avoid ice creams, pastry desserts and chocolate consumption. Eat rusks or simple biscuits for breakfast. Sugar should be avoided.
14. Alcohol consumption is not recommended. Avoid sugar drinks, industrial teas and fruit juices with or without added sugar.
15. 2-3 cups of coffee per day are allowed.
16. Recommended cooking methods: grilled, grilled, steamed, boiled, roasted, in foil, in the oven. To season the dishes, we recommend to use vegetable or meat broth, tomato sauce, vegetables, spices such as basil, oregano, saffron, marjoram, pepper, garlic.
17. Limit use of salt and avoid all foods rich in salt (pickled or canned foods).
18. It is recommended to do regular moderate-intensity exercise (≥ 30 minutes a day)

1.3 Diet with weekly food frequency and total energy intake of 1700 kcal/day in overweight women

1. Limit meat consumption twice a week and prefer white meat (i.e. poultry or lean pork), deprived of skin and visible fat. Limit red meat consumption once a week. Avoid offal consumption (i.e. brain and kidney).
   Portion: 100 g
2. Increase fish products consumption at least 2-4 times a week; prefer fish rich in omega-3 fatty acids (sardines, herring, anchovies, salmon). Crustaceans and mollusks are not contraindicated because they contain moderate amounts of cholesterol which are lower than meat and cheese.

	**Portion**
Lobster, crab, shrimp, mussel, squid, cuttlefish, oyster, boga, octopus, sea snails, anchovy, cod, hake, soaked cod, stockfish, sea bream, sole, murmur, snapper, grouper, corvina, dogfish, trout, pagro , croaker, moray, bass, breed, turbot, anglerfish, tench, pike.	150 g
fresh salmon, fresh tuna, carp, mullet, swordfish, mackerel, sardine, herring, sturgeon, sarago, verdesca, latterini, eel.	100 g

3. Limit processed meat consumption once a week and prefer hams deprived of visible fat and turkey. Avoid sausages, bacon, salami and corned beef consumption.
   Portion: raw ham 90 g or
   cooked ham 100 g or
   turkey 120 g.
4. Limit low-fat cheeses and dairy products consumption twice a week. Grated Parmesan should be limited to 1 tablespoon per day.
   Low-fat cheese portion: 50 g
5. We recommend daily use of partially skimmed or skimmed milk or yogurt.
   Milk portion: 150 ml or
   Yogurt portion: 120 g
6. Limit eggs consumption to twice eggs a week.
7. Avoid butter, margarine, lard, palm oil and foods that contain them. Use daily extra virgin olive oil daily. Avoid also fried foods, mayonnaise and pre-cooked frozen food.
   Extra virgin olive oil portion: 3 spoons a day.
8. Eat daily bread, pasta, rice, or other cereals, preferably whole meal. Avoid consumption of fresh egg pasta, oil or milk bread and focaccia.
   Bread portion: 100 g per day (or whole meal bread: 130 g per day)
   Pasta portion: 80 g per day.
9. Consume legumes at least 2-3 times a week (beans, chickpeas, lentils, peas, broad beans).
10. Consume vegetables and vegetables of any kind daily. Potatoes are not considered vegetables; they are substitutes of bread and pasta and must be consumed no more than once a week due to the high glycemic index.
    Vegetable portion: free.
    Potatoes portion: 350 g instead of 80 g of pasta or 100 g of bread.
11. Consume fruit daily. Limit frequency of consumption of very ripe and sugary fruits (i.e. bananas, persimmons, figs, prickly pears, grapes). Avoid coconut for saturated fat content.
    Fruit portion: two per day.
    Portion of pineapple oranges, cherries, kiwi, mapo, pears, apples, plums: 150 g

Portion of apricots, watermelon, strawberries, raspberries, quinces, melon, blueberries, blackberries, medlars, peaches, grapefruit: 210 g
Fruits to be limited: bananas, persimmons, figs, prickly pears, mandarins, mangoes, pomegranates, grapes: 100 g

12. Consume a small portion of dried nuts daily (walnuts, almonds, hazelnuts, cashews, pistachios).
Nuts portion: 20 g
13. Sugar should be avoided. Avoid also ice creams, pastry desserts and chocolate consumption. Eat rusks or simple biscuits for breakfast.
Portions: 4 rusks or Whole meal rusks n.5 or simple biscuits n.4 or bread 40 g.
14. Alcohol consumption is not recommended. Avoid sugar drinks, industrial teas and fruit juices with or without added sugar.
15. 2-3 cups of coffee per day are allowed.
16. Recommended cooking methods: grilled, grilled, steamed, boiled, roasted, in foil, in the oven. To season the dishes, we recommend to use vegetable or meat broth, tomato sauce, vegetables, spices such as basil, oregano, saffron, marjoram, pepper, garlic.
17. Limit use of salt and avoid all foods rich in salt (pickled or canned foods).
18. It is recommended to do regular moderate-intensity exercise (≥ 30 minutes a day)

1.4 Diet with weekly food frequency and total energy intake of 2100 kcal/day in overweight men

1. Limit meat consumption twice a week and prefer white meat (i.e. poultry or lean pork), deprived of skin and visible fat. Limit red meat consumption once a week. Avoid offal consumption (i.e. brain and kidney).
Portion: 150 g
2. Increase fish products consumption at least 2-4 times a week; prefer fish rich in omega-3 fatty acids (sardines, herring, anchovies, salmon). Crustaceans and mollusks are not contraindicated because they contain moderate amounts of cholesterol which are lower than meat and cheese.

	Portion
Lobster, crab, shrimp, mussel, squid, cuttlefish, oyster, boga, octopus, sea snails, anchovy, cod, hake, soaked cod, stockfish, sea bream, sole, murmur, snapper, grouper, corvina, dogfish, trout, pagro , croaker, moray, bass, breed, turbot, anglerfish, tench, pike.	200 g
fresh salmon, fresh tuna, carp, mullet, swordfish, mackerel, sardine, herring, sturgeon, sarago, verdesca, latterini, eel.	150 g

3. Limit processed meat consumption once a week and prefer hams deprived of visible fat and turkey. Avoid sausages, bacon, salami and corned beef consumption.
Portion: raw ham 130 g or
cooked ham 150 g or
turkey 190 g.
4. Limit low-fat cheeses and dairy products consumption twice a week. Grated Parmesan should be limited to 1 tablespoon per day.
Low-fat cheese portion: 80 g
5. We recommend daily use of partially skimmed or skimmed milk or yogurt.
Milk portion: 200 ml or

Yogurt portion: 150 g

6. Limit eggs consumption to twice eggs a week.
7. Avoid butter, margarine, lard, palm oil and foods that contain them. Use daily extra virgin olive oil daily. Avoid also fried foods, mayonnaise and pre-cooked frozen food.
   Extra virgin olive oil portion: 4 spoons a day.
8. Eat daily bread, pasta, rice, or other cereals, preferably whole meal. Avoid consumption of fresh egg pasta, oil or milk bread and focaccia.
   Bread portion: 140 g per day (or whole meal bread: 180 g per day)
   Pasta portion: 100 g per day.
9. Consume legumes at least 2-3 times a week (beans, chickpeas, lentils, peas, broad beans).
10. Consume vegetables and vegetables of any kind daily. Potatoes are not considered vegetables; they are substitutes of bread and pasta and must be consumed no more than once a week due to the high glycemic index.
    Vegetable portion: free.
    Potatoes portion: 400 g instead of 100 g of pasta or 140 g of bread.
11. Consume fruit daily. Limit frequency of consumption of very ripe and sugary fruits (i.e. bananas, persimmons, figs, prickly pears, grapes). Avoid coconut for saturated fat content.
    Fruit portion: two per day.
    Portion of pineapple oranges, cherries, kiwi, mapo, pears, apples, plums: 150 g
    Portion of apricots, watermelon, strawberries, raspberries, quinces, melon, blueberries, blackberries, medlars, peaches, grapefruit: 210 g
    Fruits to be limited: bananas, persimmons, figs, prickly pears, mandarins, mangoes, pomegranates, grapes: 100 g
12. Consume a small portion of dried nuts daily (walnuts, almonds, hazelnuts, cashews, pistachios).
    Nuts portion: 20 g
13. Sugar should be avoided. Avoid also ice creams, pastry desserts and chocolate consumption. Eat rusks or simple biscuits for breakfast.
    Portions: 6 rusks or whole meal rusks n.7 or simple biscuits n.5 or bread 70 g.
14. Alcohol consumption is not recommended. Avoid sugar drinks, industrial teas and fruit juices with or without added sugar.
15. 2-3 cups of coffee per day are allowed.
16. Recommended cooking methods: grilled, grilled, steamed, boiled, roasted, in foil, in the oven. To season the dishes, we recommend to use vegetable or meat broth, tomato sauce, vegetables, spices such as basil, oregano, saffron, marjoram, pepper, garlic.
17. Limit use of salt and avoid all foods rich in salt (pickled or canned foods).
18. It is recommended to do regular moderate-intensity exercise ($\geq$ 30 minutes a day)

2.4 Statistical analysis

Statistical analysis was performed using IBM SPSS Statistics, Release Version 25.0 (SPSS, Inc., 2017, Chicago, IL, www.spss.com). Kolmogorov-Smirnov Analysis was performed to test the normality of variables. Results of continuous variables were expressed as median and interquartile range. For ordinal and nominal variables, were used contingency tables indicating frequency and percentage in the population. For the comparison of continuous variables between different groups of patients were used non-parametric tests of Kruskal–Wallis or Mann–Whitney when appropriate. Nominal variables were examined with the Pearson chi square (X^2) test and with Spearman's rank correlation index for the correlation with continuous variables. Intergroup comparisons were adjusted for multiple comparisons with Bonferroni correction. Multivariate analysis in baseline condition were set as follow: dependent variables were baseline TC, HDL-C, LDL-C and TG and predictors were sex (male=0 and female=1), Age (0= <45y and 1=≥ 45 y), BMI (0= < 25 kg/m2) and (1= ≥ 25 kg/m2), smoking habit (0= no/past smoker and 1= current smoker) and ethanol daily intake (0= < 10g/die and 1= ≥ 10g/die). Multivariate analysis for lipid variations were set as follow: Dependent variables were variation in TC, HDL-C, LDL-C and TG and predictors were sex (male=0 and female=1), Age (0= <45y and 1=≥ 45 y), BMI (0= < 25 kg/m2) and (1= ≥ 25 kg/m2), smoking habit (0= no/past smoker and 1= current smoker) and ethanol daily intake (0= < 10g/die and 1= ≥ 10g/die).

Supplementary table S2. General characteristics of patients according to intervention types and gender.

VARIABLE	DIET ALONE	DIET+NUT	P-value†, ¶
**Age** [years: mean±SD; median; IQ range]	F: 51±16; 54 (40, 61)<br>M: 48±14; 48 (40, 58)	F: 55±13; 57 (49, 65)<br>M: 46±12; 45 (37, 54)	*p*<0.001 for F vs M in both intervention groups
**Weight** [kg: mean±SD; median; IQ range]	F: 63.4±10.9; 62.0 (55.0, 70.0)<br>M: 80.9±15.0; 81.0 (71.5, 88.5)	F: 63.9±13.4; 62.0 (55.0, 70.0)<br>M: 77.4±11.7; 77.0 (72.0, 83.0)	*p*<0.001 for F vs M in both intervention groups
**BMI** [kg/m^2: mean±SD; median; IQ range]	F: 24.4±4,3; 23.9 (21.2, 27,0)<br>M: 26.3±4,1; 25.7 (23.4, 28,4)	F: 24.4±4.1; 23.6 (22.0, 26.5)<br>M: 25.4±3.2; 24.9 (23,7, 26.9)	*p*<0.001 for F vs M in both intervention groups
**SBP** [mm/Hg: mean±SD; median; IQ range]	F: 126±2; 126 (125, 128)<br>M: 126±3; 126 (124, 128)	F: 127±2; 127 (125, 128)<br>M: 127±2; 127 (125, 128)	NS
**DBP** [mm/Hg: mean±SD; median; IQ range]	F: 85±3; 84 (82, 87)<br>M: 85±3; 85 (83, 87)	F: 85±3; 85 (83, 88)<br>M: 84±3; 85 (82, 86)	NS
**Smoking habits** [Current: n; %]	F: 25/117 (21.6%)<br>M: 47/90 (52.2%)	F: 35/90 (21.0%)<br>M: 35/111 (31.5%)	*p*<0.001 for F vs M in diet alone group
**Alcohol intake** [Excessive: n; %]	F: 19 (16,2%)<br>M: 33 (36,7%)	F: 32 (18,9%)<br>M: 30 (27%)	*p*<0.001 for F vs M in both intervention groups

Abbreviations: M=male, F=female, BMI=body mass index, NUT= Lipid lowering nutraceutical

† P-value at Mann-Whitney test and Pearson's chi square test for continuous and nominal data respectively.

¶ No statistically significant difference between diet alone and diet plus NUT patients.

Supplementary table S3. Baseline lipid profile in all patients and divided in subgroups according to sex, age, BMI, smoking habits, alcohol consumption and types of lifestyle intervention.

	TC	HDL-C	LDL-C	TG
**All patients**				
**Sex***				
F	270 (248; 298) †	62 (52; 75) †	179 (161; 202)	113 (84; 160) †
M	256 (229; 277)	47 (39; 55)	171 (152; 198)	181 (116; 283)
**Age***				
<45 years	258 (233; 285)	52 (41; 62) †	179 (155; 200)	131 (87; 228)
≥45 years	266 (242; 293)	57 (47; 71)	177 (158; 199)	134 (95; 197)
**BMI***				
<25 kg/m^2	263 (242; 287)	60 (49; 73) †	176 (157; 198)	116 (82; 169) †
≥25 kg/m^2	263 (238; 296)	50 (41; 61)	180 (156; 206)	163 (108; 261)
**Smoking habits***				
Never/Past	264 (242; 292)	56 (46; 69)	178 (158; 203)	131 (92; 206)
Current	259 (232; 282)	53 (42; 66)	178 (152; 195)	137 (99; 230)
**Alcohol consumption***				
Absent/moderate	262 (238; 292)	56 (44; 70)	177 (155; 199)	128 (91; 200)
Elevate	265 (245; 286)	54 (46; 64)	178 (159; 200)	143 (97; 229)
**Lifestyle intervention***				
Diet alone	259 (241; 285)	54 (42; 66)	176 (154; 194)	142 (96; 232)
Diet + NUT	265 (240; 294)	56 (47; 69)	181 (158; 203)	128 (89; 195)

Data are median (IQR). Abbreviations: M=male, F=female, BMI=body mass index, NUT= Lipid lowering nutraceutical

* Independent pairwise comparisons among subgroup terms with Mann-Whitney U test adjusted for multiple comparisons (Bonferroni).

† The difference is significant at the 0.001 level between subgroup terms.

Supplementary table S4. Multivariate analysis on baseline lipid profile in all 487 patients

**VARIABLE and PREDICTORS**	β	SE	*p*-value ¶	r^2	F (*p*-value) †
**TC**				0.073	7.577 (0.0001)
Male sex	-19,2	4,0	<0,0001		
Age ≥ 45 y	4,7	4,0	0,243		
BMI ≥ 25 kg/m^2	4,8	3,7	0,196		
Current smoker	-6,4	4,3	0,137		
Ethanol ≥ 10g/die	7,1	4,1	0,080		
**HDL-C**				0.236	29.909 (0,0001)
Male sex	-15,4	1,8	<0.0001		
Age ≥ 45 y	2,0	1,8	0,278		
BMI ≥ 25 kg/m^2	-8,8	1,6	<0.0001		
Current smoker	-1,5	1,9	0,432		
Ethanol ≥ 10g/die	3,3	1,8	0,068		
**LDL-C**				0,021	1.807 (0,110)
Male sex	-9,1	3,8	0,016		
Age ≥ 45 y	-1,9	3,8	0,628		
BMI ≥ 25 kg/m^2	5,8	3,5	0,101		
Current smoker	-3,4	4,01	0,411		
Ethanol ≥ 10g/die	3,4	3,8	0,373		
**TG**				0,136	14,905 (0,0001)
Male sex	80,5	13,5	<0,0001		
Age ≥ 45 y	11,4	13,7	0,406		
BMI ≥ 25 kg/m^2	55,3	12,4	<0,0001		
Current smoker	6,7	14,4	0,643		
Ethanol ≥ 10g/die	-10,8	13,6	0,430		

Dependent variables were baseline TC, HDL-C, LDL-C and TG and predictors were sex (male=0 and female=1), Age (0= <45y and 1=≥ 45 y), BMI (0= < 25 kg/m^2) and (1= ≥ 25 kg/m^2), smoking habit (0= no/past smoker and 1= current smoker) and ethanol daily intake (0= < 10g/die and 1= ≥ 10g/die).

Abbreviation: β=angular coefficient, SE=standard error, r^2=square correlation coefficient, F= F-value
Abbreviations: BMI=body mass index

¶ *p*-values for predictors

†*p*-value for model fitting significance

Supplementary table S5. Multivariate analysis between baseline and follow-up of lipid profile in 207 patients on diet alone treatment.

**VARIABLES and PREDICTORS**	β	SE	*p*-value ¶	r^2	F (*p*-value) †
**Variation in TC**				0.030	1,235 (0,294)
Male sex	10,5	5,8	0,071		
Age ≥ 45 y	2,5	5,7	0,661		
BMI ≥ 25 kg/m^2	-1	5,3	0,847		
Current smoker	-4,5	6	0,452		
Ethanol ≥ 10g/die	5,1	5,7	0,372		
**Variation in HDL-C**				0.045	1.768 (0,121)
Male sex	1,4	1,6	0,406		
Age ≥ 45 y	1,3	1,6	0,402		
BMI ≥ 25 kg/m^2	2,0	1,5	0,175		
Current smoker	1,6	1,6	0,339		
Ethanol ≥ 10g/die	-3,2	1,6	<u>0,041</u>		
**Variation in LDL-C**				0,068	2.440 (0.037)
Male sex	11,3	4,9	<u>0,024</u>		
Age ≥ 45 y	2,6	4,9	0,589		
BMI ≥ 25 kg/m^2	-0,6	4,5	0,893		
Current smoker	-6,3	5,1	0,225		
Ethanol ≥ 10g/die	7,2	4,8	0,142		
**Variation in TG**				0.049	2.024 (0.077)
Male sex	-58,7	28.0	<u>0,037</u>		
Age ≥ 45 y	9,6	27,5	0,728		
BMI ≥ 25 kg/m^2	-38,7	25,5	0,131		
Current smoker	-4,7	29,0	0,872		
Ethanol ≥ 10g/die	32,7	27,5	0,237		

Dependent variables were variation in TC, HDL-C, LDL-C and TG and predictors were sex (male=0 and female=1), Age (0= <45y and 1=≥ 45 y), BMI (0= < 25 kg/m^2) and (1= ≥ 25 kg/m^2), smoking habit (0= no/past smoker and 1= current smoker) and ethanol daily intake (0= < 10g/die and 1= ≥ 10g/die).

Abbreviation: β=angular coefficient, SE=standard error, r^2=square correlation coefficient, F= F-value
Abbreviations: BMI=body mass index

¶ *p*-values for predictors

†*p*-value for model fitting significance

Supplementary table S6. General characteristics and lipid parameters of 280 patients on diet plus NUT treatment.

**Variable**		AP: MonK (3mg) + BBR (500mg)	PS (1.6 g)	N5: MonK (10 mg)	BBK: MonK (10 mg) + BBR (500mg)	BERA: MonK (3 mg) + BBR (500mg)	LSB: MonK (3mg) + BBR (500mg)	W3: PUFA-W3 (3g)	AP+W3: MonK (3mg) + BBR (500mg) + PUFA-W3 (3g)	Others
**Sex** [F/M: n; %]		74 (64.3%) / 41 (35.7%)	8 (80.0%) / 2 (20.0%)	22 (75.9%) / 7 (24.1%)	19 (63.3%) / 11 (36.7%)	8 (88.9%) / 1 (11.1%)	9 (69.2%) / 4 (30.8%)	7 (19.4%) / 29 (80.6%)	7 (70.0%) / 3 (30.0%)	13 (56.5%) / 10 (43.5%)
**Age** [years: mean±SD; median; IQ range]		51±13; 52 (43, 62)	49±9; 51 (46, 53)	57±15; 59 (53, 66)	53±10; 55 (44, 61)	59±11; 55 (52, 65)	53±11; 52 (44, 62)	49±13; 50 (39, 58)	50±15; 49 (43, 59)	49±18; 49 (39, 61)
**Weight** [kg: mean±SD; median; IQ range]		68.43±15.45; 67 (56, 75)	69±7.32; 66.5 (65, 73)	68.31±14.24; 69.85 (58, 75)	68.82±10.97; 68.7 (59.9, 74.8)	59.67±4.15; 60 (57, 60)	64.46±11.03; 65 (60, 72)	77.36±12.65; 77 (70, 83)	69.3±15.41; 65 (56, 84)	66.65±17.37; 70 (56.5, 75)
**BMI** [kg/m^2: mean±SD; median; IQ range]		24.6±3.8; 24.5 (22.1, 26.5)	24.8±2.7; 25.1 (24.6, 26.4)	25.3±4.6; 24 (22.4, 28.1)	24.7±2.2; 24.5 (23.4, 26.5)	23±1; 23.4 (22.4, 23.4)	24.3±4.7; 23.5 (22.3, 26.6)	26±3.7; 25.2 (23.7, 27.5)	24.5±4.8; 22.6 (22, 27.7)	24.3±4.6; 24 (22.1, 27.3)
**SBP** [mm/Hg: mean±SD; median; IQ range]		126±2; 126 (125, 128)	126±2; 126 (125, 128)	127±3; 127 (125, 129)	127±2; 128 (126, 129)	127±2; 128 (126, 129)	127±2; 128 (125, 129)	127±2; 127 (125, 129)	127±2; 127 (125, 128)	127±2; 126 (125, 129)
**DBP** [mm/Hg: mean±SD; median; IQ range]		85±3; 85 (82, 87)	84±2; 84 (83, 85)	85±3; 85 (83, 86)	86±2; 86 (85, 88)	85±3; 84 (83, 87)	85±3; 86 (83, 88)	85±3; 86 (83, 87)	84±3; 84 (81, 87)	85±3; 85 (82, 88)
**Risk SCORE** [%: mean±SD; median; IQ range]		1.9±2.5%; 0.7% (0.3, 2.9%)	0.9±1%; 0.6% (0.4, 1.2%)	2.9±4.5%; 1.3% (0.7, 4.6%)	1.4±1.3%; 1.2% (0.5, 1.5%)	2.7±3.3%; 2% (0.5, 3.9%)	1.8±1.8%; 1.1% (0.5, 2.8%)	2.4±3.4%; 0.9% (0.2, 2.1%)	1.5±2.4%; 0.7% (0.2, 1.4%)	2.3±3.9%; 0.7% (0.3, 2.3%)
**Smoking habits** [Never + Past/Current: n; %]		87 (75.7%) / 28 (24.3%)	7 (70.0%) / 3 (30.0%)	25 (86.2%) / 4 (13.8%)	27 (90.0%) / 3 (10.0%)	8 (88.9%) / 1 (11.1%)	10 (76.9%) / 3 (23.1%)	24 (66.7%) / 12 (33.3%)	10 (100.0%) / 0 (0.0%)	16 (69.6%) / 7 (30.4%)
**Alcohol intake** [No + Moderate/Excessive: n; %]		77 (67.5%) / 37 (32.5%)	9 (90.0%) / 1 (10.0%)	22 (75.9%) / 7 (24.1%)	24 (80.0%) / 6 (20.0%)	7 (77.8%) / 2 (22.2%)	12 (92.3%) / 1 (7.7%)	25 (71.4%) / 10 (28.6%)	10 (100.0%) / 0 (0.0%)	19 (82.6%) / 4 (17.4%)
**TC** [mg/dL: median; IQR]	Baseline	276 (256, 298)	240 (234, 255)	253 (234, 277)	255 (235, 272)	275 (254, 276)	304 (282, 310)	242 (211, 268)	293 (251, 310)	258 (230, 305)
	F/U	230 (209, 253)	217 (201, 238)	222 (207, 236)	206 (185, 232)	221 (213, 245)	247 (214, 263)	222 (197, 254)	240 (222, 257)	235 (191, 258)
	Variation	-41 (-66, -20), -15,2%	-34 (-41, -19), -13,1%	-30 (-48, -20), -11,3%	-54 (-60, -39), -20,7%	-54 (-63, -41), -22,2%	-40 (-84, -34), -15,4%	-15 (-37, 5), -6%	-44 (-68, -22), -14,9%	-22 (-64, -10), -8,2%
**HDL-C** [mg/dL: median; IQR]	Baseline	58 (49, 72)	61 (57, 62)	59 (48, 75)	63 (48, 78)	58 (56, 73)	57 (47, 83)	40 (33, 47)	56 (52, 59)	60 (47, 62)
	F/U	58 (48, 70)	53 (52, 61)	58 (46, 73)	61 (51, 73)	60 (50, 70)	57 (52, 65)	39 (36, 52)	58 (50, 61)	56 (51, 70)
	Variation	0 (-8, 6), 0%	-8 (-9, 0), -13,6%	-1 (-6, 1), -2,2%	-1 (-5, 4), -0,4%	-2 (-11, 6), -3,8%	3 (-5, 5), 3,4%	3 (-1, 7), 5,3%	1 (-5, 6), 0,6%	3 (-5, 10), 4%

**LDL-C** [mg/dL: median; IQR]	Baseline	189 (165, 206)	165 (152, 175)	174 (159, 195)	164 (148, 174)	180 (171, 203)	197 (183, 212)	158 (130, 178)	215 (199, 222)	186 (151, 219)
	F/U	147 (130, 161)	139 (126, 155)	142 (124, 154)	121 (100, 142)	149 (134, 164)	167 (129, 183)	142 (121, 169)	149 (138, 175)	154 (124, 169)
	Variation	-38 (-62, -20), -19,4%	-23 (-48, -17), -13,3%	-23 (-41, -14), -14,7%	-47 (-52, -34), -28%	-44 (-54, -42), -25,4%	-44 (-79, -16), -19,7%	-11 (-40, 9), -7,2%	-51 (-79, -17), -25,1%	-22 (-51, -2), -12,3%
**TG** [mg/dL: median; IQR]	Baseline	124 (84, 166)	104 (75, 126)	121 (92, 157)	110 (81, 178)	124 (107, 154)	119 (95, 157)	265 (196, 345)	158 (103, 245)	123 (88, 195)
	F/U	104 (82, 149)	90 (80, 118)	111 (88, 128)	101 (81, 142)	100 (92, 111)	119 (75, 131)	187 (136, 230)	144 (104, 180)	102 (79, 125)
	Variation	-11 (-38, 13), -9,4%	5 (-8, 15), 5,8%	-12 (-39, 1), -14,1%	-8 (-58, 30), -9%	-26 (-33, 3), -22,6%	-23 (-50, 9), -23%	-66 (-148, -18), -22,6%	-25 (-68, -9), -25,6%	-24 (-71, -4), -20,5%

Abbreviations: M=male, F=female, BMI=body mass index, IQR, interquartile range, SBP=systolic blood pressure, DBP=diastolic blood pressure, MonK= MonK= monacolin K, MonK + BBR= berberine, PS= Plant Sterols, PUFA-W3= Ω3 - polyunsaturated fatty acid
